# Encapsulation of Babchi Oil in Cyclodextrin-Based Nanosponges: Physicochemical Characterization, Photodegradation, and In Vitro Cytotoxicity Studies

**DOI:** 10.3390/pharmaceutics10040169

**Published:** 2018-09-26

**Authors:** Sunil Kumar, Pooja, Francesco Trotta, Rekha Rao

**Affiliations:** 1 Department of Pharmaceutical Sciences, Guru Jambheshwar University of Science and Technology, Hisar 125001, Haryana, India; sunilkundu450@gmail.com; 2 Department of Biotechnology, Indian Institute of Technology, Roorkee 247667, India; poojasihag1592@gmail.com; 3 Department of Chemistry, University of Torino, 10135 Turino, Italy; francesco.trotta@unito.it

**Keywords:** essential oil, *Psoralea corylifolia*, cytotoxicity, encapsulation, solubilisation

## Abstract

Babchi (*Psoralea corylifolia*) oil is an important essential oil used in several traditional medicines to cure various disorders. This phytotherapeutic agent possesses a number of pharmacological activities including antibacterial, antifungal, antioxidant, anti-inflammatory, immunomodulatory, and antitumor factors. However, volatile nature, poor stability, and solubility of babchi oil (BO) restrict its pharmaceutical applications. Therefore, the aim of the present work was to encapsulate this oil in β-cyclodextrin nanosponges (NS) in order to overcome the above limitations. To fabricate nanosponges, β-cyclodextrin was cross-linked with diphenyl carbonate in different molar ratios viz. 1:2, 1:4, 1:6, 1:8, and 1:10. The blank nanosponges were loaded with BO using the freeze-drying method. The particle size of the BO loaded nanosponges was found to lie between 200 and 500 nm with low polydispersity index. Furthermore, the zeta potential, the Fourier transform infrared spectroscopy, X-ray diffraction, thermal analysis, and electron microscopy were carried out for characterization of BO nanosponges. Results obtained from spectral analysis ascertained the formation of inclusion complexes. Additionally, solubilisation efficiency of BO was checked in distilled water and found enhanced by 4.95 times with optimized β-cyclodextrin nanosponges. The cytotoxicity study was carried out by the MTT assay using HaCaT cell lines. A significant improvement in photo-stability of essential oil was also observed by inclusion innanosponges. Lastly, the optimized formulation was tested for antibacterial activity using *Staphylococcus aureus*, *Pseudomonas aeruginosa*, and *Escherichia coli*. Therefore, encapsulation of BO in nanosponges resulted in efficacious carrier system in terms of solubility, photo-stability, and safety of this oil along with handling benefits.

## 1. Introduction

Encapsulation strategies play a vital role for the delivery of poorly soluble, unstable, or toxic moieties. Enhancing their encapsulation efficiency using carrier systems can help achieve better therapeutic efficacy due to a reduction in side effects. Therefore, newer encapsulation techniques are explored using natural polymers nowadays.

Naturally occurring polysaccharides act as attractive polymers for drug delivery systems due to their high biodegradability, biocompatibility, and lower cost [1]. One of the most extensively used natural polysaccharide includes cyclodextrins. Cyclodextrins possess the ability to encapsulate guest molecules inside their internal cavity, which leads to modification of the physico-chemical features of the host molecules like the physical state, solubility, stability, and bioavailability [2,3]. However, cyclodextrins are unable to form inclusion complexes with hydrophilic or high-molecular-weight molecules [4]. In the last few years, a nanosponge has been proposed as an advanced drug delivery system involving the reaction of cyclodextrin with a suitable cross-linking agent for encapsulation of difficult-to-formulate moieties [5,6,7,8]. These are hyper cross-linked nanoporous structures reported to form inclusion and non-inclusion complexes with a variety of drugs to enhance their solubility, stability, permeability, cytotoxicity, and other such drug delivery features [9].

Essential oils composed of lipophilic and highly volatile secondary plant metabolites represent a “natural” alternative in pharmaceutical, cosmetic, food, and agriculture fields due to their antiviral, antimicrobial, antifungal, insecticidal, nematicidal, and antioxidant properties [10]. An important essential oil obtained from *Psoralea coryfolia*(belonging to family Fabaceae) is babchi oil (BO), which is a vital phytotherapeutic agent reported for the treatment of psoriasis [11]. An anti-psoriatic effect of BO is attributed to psoralen, bakuchiol, and isopsoralen content, which are the major constituents of this oil [11,12]. These chief constituents (furocoumarins) collectively inhibit DNA synthesis, which causesa reduction in cell proliferation [13]. In addition, the BO also possesses numerous activities like antifungal, antibacterial, antiviral, antitumor, cytotoxic, antioxidant, antidepressant and stimulant [11]. However, this essential oil is found to have poor aqueous solubility, stability, and is volatile in nature [14]. These drawbacks limit the practical use of this oil in spite of its numerous beneficial effects. Faiyazuddin et al. fabricated and evaluated solid lipid nanoparticles of BO. However, poor stability, sterilization difficulties, and low drug loading limits the use of solid lipid nanoparticles [15]. Unlike these, cyclodextrin nanosponges possess larger cavities for host molecules to be entrapped in the nano pores due to a crosslinking network of a polymer and a cross linker. In addition, these nano systems have also been proposed as stable carriers for the entrapment of a variety of therapeutic agents [16,17,18,19]. However, various phyto-constituents like resveratrol [20], γ-oryzanol [18], curcumin [21], quercetin [19], rutin, phloridzin, and chlorogenic acid [22] have been successfully encapsulated in cyclodextrin nanosponges.There are no reports on the encapsulation of essential oils using cyclodextrin nanosponges. Based on the facts above, the purpose of the present study was to evaluate the nanosponge as an encapsulating agent for babchi essential oil found in *Psoralea coryfolia* seeds. In this preliminary investigation, β-cyclodextrin (β-CD) nanosponges were purposely tailored for the formulation of this essential oil with poor water solubility. Prepared nanosponges may solubilize BO by complexation and improve its stability. The physico-chemical characterization of the BO nanosponge formulations was carried out by thermogravimetry analysis (TGA), X-ray powder diffraction (XRPD), and Fourier transform infra-red (FTIR) spectroscopy. The fabricated nanosponges (NS) were also characterized regarding topography and a microstructure using a field emission scanning electron microscopy (FE-SEM) and transmission electron microscopy (TEM). In addition, particle size analysis, zeta potential, and a polydispersity index were also investigated for BO loaded nano-formulations. The safety of the optimized nanosponges was assessed with an MTT assay using HaCaT (human epidermal keratinocyte) cell line. Furthermore, in vitro antibacterial studies were explored to compare the antibacterial efficacy of the optimized nanosponges against plain BO. The photo-degradation of BO and BO loaded nanosponges upon UVA irradiation were also carried out.

## 2. Materials and Methods

### 2.1. Materials

The BO was obtained as a generous gift sample from Pukhraj Herbals (Mandsaur, India). β-cyclodextrin (β-CD) was procured from JayChemMarketing (Mumbai, India). Diphenyl carbonate (DPC) was purchased from Sigma-Aldrich (Milan, Italy). All other reagents and chemicals used were of an analytical grade. Double distilled water was used throughout the studies.

### 2.2. Gas Chromatography Mass Spectrometry of the Babchi Oil (BO)

Gas chromatography mass spectrometry (GC-MS) analysis was carried out using a GC-2010 gas chromatography (Shimadzu Corp., Kyoto, Japan) equipped with a GCMS-QP2010 Plus along with thermal desorption system TD 20 (Shimadzu Corp., Kyoto, Japan). A sample (0.2 μL) was injected into the split injector with a split ratio of 1:100. The oven temperature was kept at 50 °C for 2 min, increased to 210 °C at a rate of 3 °C/min and held for 1 min, and then increased to 280 °C at a rate of 8 °C/min and held for 6 min. The injector temperature was 260 °C while the ion source temperature and interface temperature were 230 °C and 270 °C, respectively. The identification of constituents was established in comparison of their mass spectra. The peak area measurement (expressed in an area percentage) was used for quantitative analysis of each component of BO.

### 2.3. Synthesis of Nanosponges

Cyclodextrin-based nanosponges were prepared using a previously reported procedure [23,24]. Different molar ratios of β-CD and DPC (1:2, 1:4, 1:6, 1:8, and 1:10) were used for the preparation of BO nano-carriers.

Finely homogenized anhydrous β-CD and DPC were gradually heated from 90 to 100 °C under magnetic stirring for 6 h. Then, crystals of phenol formed at the neck of the flask were carefully removed. The reaction mixture was allowed to cool at room temperature. The solid obtained was repeatedly washed with double distilled water in order to remove unreacted β-CD and subsequently purified by a soxhlet extraction process using acetone to separate the unreacted DPC and phenol from the product. After every washing of acetone, a test for phenol was performed using ferric chloride solution. The nanosponges were dried at 40 °C in a hot air oven for 2 h and stored at room temperature in a dessicator until further use [24].

### 2.4. Solubilization Efficiency of Nanosponges

In order to examine the solubilization enhancement capacity, the solubilization efficiency of the BO in β-CD and all the batches of nanosponges (NS2, NS4, NS6, NS8, and NS10) were investigated [19,21]. An excess amount of the BO (50 mg) was taken with a fixed amount (20 mg) of blank nanosponges in 20 mL of double distilled water in a volumetric flask. The volumetric flasks were allowed to shake on a mechanical shaker at an ambient temperature for 24 h. After equilibrium, the obtained suspensions were centrifuged at 10,000 rpm for 10 min to assort a colloidal supernatant and free BO. Dimethyl sulfoxide (10 mL) was added to supernatants in order to extract the encapsulated BO from the nanosponges. After 2 h, the colloidal supernatant solutions were examined by a UV spectrophotometer (Varian Cary-5000, Christ, The Netherlands) at 265 nm using a calibration curve of BO. The experiments were performed in triplicate.

### 2.5. Preparation of Babchi Oil-Loaded β-Cyclodextrin Nanosponges

BO was loaded in the blank nanosponges by using a freeze-drying technique, which was previously reported [25]. Accurately weighed quantities (1 gm) of blank nanosponges were dispersed in 50 mL of double distilled water using a magnetic stirrer, then an excess amount of BO was added, and the mixture was sonicated for 10 min and kept for 24 h under stirring. The suspensions were centrifuged at 3000 rpm for 10 min to separate the un-complexed BO as a residue below the colloidal supernatant. The supernatant obtained was freeze-dried using a lyophilizer (Alpha 2-4 LD Plus CHRIST, Osterode, Germany) at −81 °C temperature and operating pressure of 0.0010 mbar. The dried powder was stored in a desiccator. BO-loaded NS formulations obtained were named asBONS2, BONS4, BONS6, BONS8, and BONS10 depending on the ratio of β-CD and DPC used in the fabrication of nanosponges [19].

### 2.6. Evaluation of Nanosponges

#### 2.6.1. Determination of the BO Content in the Nanosponges

A weighed amount of all types of BO-loaded NSs was dispersed in dimethylsulfoxide (DMSO), sonicated for 10 min to break the nanosponge complex, and diluted using DMSO. Furthermore, the prepared samples were analysed by using a UV spectrophotometer at 265 nm to ascertain the BO content in nanosponges. The encapsulation efficiency and essential oil loading capacity were calculated using the following equations.

(1) $$\mathrm{Babchi}\;\mathrm{oil}\;\mathrm{loading}(\%)\;=\frac{\mathrm{weight}\;\mathrm{of}\;\mathrm{essential}\;\mathrm{oil}\;\mathrm{in}\;\mathrm{nanosponges}}{\mathrm{weight}\;\mathrm{of}\;\mathrm{nanosponges}\;}\times 100$$

(2) $$\mathrm{Entrapment}\;\mathrm{efficiency}(\%)\;=\frac{\mathrm{weight}\;\mathrm{of}\;\mathrm{essentail}\;\mathrm{oil}\;\mathrm{in}\;\mathrm{nanosponges}}{\mathrm{weight}\;\mathrm{of}\;\mathrm{essential}\;\mathrm{oil}\;\mathrm{feed}\;\mathrm{initially}}\times 100$$

#### 2.6.2. Determination of Size, Polydispersity Index, and Zeta Potential of the Nanosponges

The size and polydispersity indices of the all NSs were determined by using dynamic light scattering with a Malvern Zetasizer (Malvern Instruments Ltd., Worcestershire, UK). The samples were suitably diluted with filtered double distilled water before each measurement. Zeta potential measurements were made using an additional electrode in the same instrument. For the zeta potential determination, the instrument was operated at a constant temperature of 25 °C using a clear disposable zeta cell. Twelve measurements were carried out and their average was expressed.

Based on the results of the encapsulation and solubilisation efficiency, the formulation BONS4 was selected for further FTIR, TGA, XRPD, FE-SEM, and TEM.

#### 2.6.3. Fourier Transform Infrared Spectroscopy

Fourier transform infrared spectra were recorded in the spectral range from 4000 to 650 cm^−1^ using a Perkin Elmer Spectrum (Waltham, MA, USA), BX II instrument in the attenuated total reflectance (ATR) mode with a diamond crystal using 45 scans per spectrum and a resolution of 2 cm^−1^.

#### 2.6.4. Thermal Analysis

Thermal analysis was performed using EXSTAR TG/DTA 6300 (Thermo gravimetric/Differential Thermal Analyzer (TG/DTA) (SII 6300 EXSTAR, Tokyo, Japan)). A heating rate of 10 °C/min was employed in a temperature range from 5 to 500 °C. The standard aluminium pans were used. Alumina powder was used as reference standard. About 10 mg of the sample was placed on the aluminium pan and subjected to the above mentioned program under a nitrogen atmosphere with 200 mL/min speed. Thermal analysis was performed to understand inclusion of BO in nanosponges. The experiments were carried out in triplicate.

#### 2.6.5. X-ray Powder Diffraction

In order to explore the host-guest interaction and structure of the resulting nanocomplexes, an X-ray powder diffraction study was carried out. Plain β-CD, blank nanosponges, and BO loaded nanosponges were subjected to XRPD using a Powder X-ray Diffractometer (Bruker D8 Advance, Karlsruhe, Germany). Diffraction profiles were analysed at a 2θ of 10° to 80° sequential collection. The step time was 0.5 s and the time of acquisition was 1 h.

#### 2.6.6. Surface Morphology

##### Field Emission Scanning Electron Microscopy

The morphology of the nanosponges and BO-loaded nanosponges was observed under FE-SEM (Carl Zeiss UltraPlus, Oberkochen, Germany). The samples were sprinkled on a double-sided carbon adhesive tape stuck to an aluminium stub and then metallized with a thin film (10 Å) of gold under an argon atmosphere for 90 s to minimize the charging effects.

##### Transmission Electron Microscopy

The morphology of the blank nanosponges and BONS4 was also observed under TEM (FEI Tecnai G^2^ 20 S-Twin, Hillsboro, OR, USA). One drop of formulation suspension was deposited on a carbon-coated copper grid and allowed to dry for contrast enhancement.

### 2.7. Cytotoxicity Studies Against the HaCaT Cell Line

The cytotoxicity of the BO and BONS4 were assessed using HaCaT cell lines. The HaCaT is a spontaneously transformed aneuploid immortal keratinocyte cell line from adult human skin [26]. These cell lines are used due to the high tendency to differentiate and anti-proliferate in vitro [27].

For cytotoxicity studies, the samples were accurately weighed and mixed to obtain the desired concentration. The mixture was dissolved in distilled DMSO and volume was made up with Dulbecco’s Modified Eagle’s medium (DMEM) and supplemented with 2% inactivated Fetal Bovine Serum (FBS) in order to obtain a stock solution (1 mg/mL). After sterilization of the stock solution by filtration, 0 µg/mL to 320 µg/mL concentration aliquots were prepared from stock solution for carrying out cytotoxic analysis.

HaCaT cells were cultured in DMEM medium and supplemented with 10% inactivated FBS, penicillin (100 IU/mL), and streptomycin (100 µg/mL) in a humidified atmosphere of 5% CO_2_ at 37 °C until confluent. The cells were dissociated with a TPVG solution (0.2% trypsin, 0.02% EDTA, 0.05% glucose in PBS).Furthermore, 50,000 cells/well of cells were seeded in a 96-well plate and incubated for 24 h at 37 °C, 5% CO_2_ incubator.

The monolayer cell culture was trypsinized and the cell count was adjusted to 1.0 × 10^5^ cells/mL using respective media containing 10% FBS. To each well (96 well microtiter plate), 100 µL of the diluted cell suspension (50,000 cells/well) was added. The plates were then incubated at 37 °C for 24 h in a 5% CO_2_ atmosphere. After an incubation period, 3-[4–dimethylthiazol-2-yl]-2, 5-diphenyl tetrazolium bromide (MTT) (5 mg/10 mL of MTT in PBS) was added to each well. After incubation of 4 h at 37 °C in a 5% CO_2_ atmosphere, the supernatant was removed. 100 µL of DMSO was added to the collected supernatant. The plates were read at 590 nm with a microplate reader (model 450). The percentage growth inhibition was determined and the concentration of the test drug needed to inhibit cell growth by 50% (IC50 values) was generated from the dose-response curves.

### 2.8. Photodegradation Study

The photo degradation of BO and BONS4 was carried out under a UV lamp (Philips 40 W TL K05, Gurgaon, India). Both samples were placed at a 10-cm distance from the UV lamp for 60 min a dark environment. The samples were exposed to a UVA wavelength range from 320 to 400 nm at room temperature (25 ± 2°C). The samples were collected at time intervals of 10 minutes and quantitatively analyzed using a UV spectrophotometer (Varian Cary 5000, Christ, The Netherlands). The experiment was performed in triplicate.

### 2.9. Antibacterial Activity

The antibacterial activity of the BO, blank nanosponges, and BO loaded nanosponges was evaluated by an agar well diffusion method using nutrient agar medium. Nutrient agar medium (30 mL) was poured into each petri plate. The test bacteria i.e., *Staphylococcus aureus ATCC* 25923 (*S. aureus*)*, Pseudomonas aeruginosa ATCC* 27853 (*P. aeruginosa*), and *Escherichia coli ATCC* 25922 (*E. coli*) (1 × 10^6^ CFU/mL) were inoculated onto the surface of medium with a sterile spreader. Afterwards, the agar medium is punched using a cork borer (diameter 6 mm). BO loaded nanosponges (equivalent to 50 mg of BO) were pipetted into the wells. The antibiotic streptomycin (STP) (10 μg/mL) was used as a positive control. The plates were allowed to incubate at 37 °C for 24 h. The diameter of the growth inhibition of all the samples surrounding the wells was examined after the incubation period. The experiment was repeated thrice and the antibacterial activity was calculated as an average value [28].

### 2.10. Statistical Analysis

All the experiments were performed in triplicate and the results were reported as mean ± standard deviation. Statistical measurements were carried out by using the GraphPad Prism version 5.01 software (GraphPad Software, San Diego, CA, USA). A One-way ANOVA (non-parametric) Kruskalwallis test was used to appraise the significant difference between the results.

## 3. Results and Discussion

Numerous efforts have been devoted to the method of the preparation and the application of nanosponges over the last decade. Amongthe various types of nanosponges, cyclodextrin-based nanosponges have received more attention and are widely studied [17].

Cyclodextrins are biodegradable, versatile compounds used to improve physicochemical and pharmaceutical properties such as solubility, stability, and bioavailability of administered drug molecules reported in literature [29]. Among the natural (α, β, γ) cyclodextrins, β-cyclodextrin has the highest complexing ability and stability with cross-linking agents. In addition, cavity dimensions, the low cost of production, and higher productive rates are other advantages offered by cyclodextrins for nanosponge preparation [30]. Various crosslinking agents like carbonyl diimmidazole, hexamethylenediisocynate, toluelyldianhydride, diisocynate, or carbonate and diphenyl carbonate have been explored in literature for nanosponge formulations [6]. In the present work, diphenyl carbonate was chosen as a cross-linker owing to its trifling acute dermal toxicity (2000 mg/kg), no skin irritancy, sensitization, and minimum lineal photo-degradation [31].

Nanosponges can be crafted using various techniques such as an ultrasound-assisted synthesis [25], a solvent evaporation technique [21], an emulsion solvent diffusion method [32], microwave assisted synthesis [33], normal solution synthesis [34], and a melt method [24].

In the present study, the melt technique has been used for preparing BO cyclodextrin nanosponges. The advantage of using this technique is that it results in a crystalline product without the use of organic solvents. Furthermore, it has been revealed that the drug loading is greater in crystalline nanosponges in comparison to paracrystalline ones [35].

Aldawsari et al. formulated and evaluated lemongrass oil-loaded ethylcellulose nanosponges using an emulsion solvent evaporation technique [36]. However, cyclodextrin nanosponges have not been explored for encapsulating essential oils. Yet these may prove as promising nanocarriers for these bioactives. Drug loading in cyclodextrin nanosponges is usually done at room temperature with afreeze drying technique after the fabrication of blank nanosponges. Hence, these nanocarriers may prove beneficial for essential oils, which are generally thermolabile, volatile, and poorly soluble. Thus, taking this into consideration, cyclodextrin nanosponges have been explored for the encapsulation of BO in the present investigation.

### 3.1. Identification of Various Compounds in BO Using GC-MS

Generally, gas chromatography is applied for qualitative analysis while for quantitative estimation, it is coupled with mass spectrometry. In the present work, GC-MS was used to identify the volatile components present in the BO sample. A large number of constituents were detected in this essential oil. Among them, bakuchiol (4-(3,7-Dimethyl-3-vinylocta-1,6-dien-1-yl)phenol) was seen as a major component with the highest percentage area (65.37%). Other main constituents included 2H-furo[2 H]-1-benzopyran-2-one (2.59%), caryophyllene oxide (2.11%), oleoyl chloride (1.70%), 2-Phenyl-4-anilino-6[1H]-pyrimidinone (1.47%), 9-Octadecenoic acid (1.29%), 2-[5-(2-Methyl-benzooxazol-7-yl)-1H-pyrazol-3-yl]-phenol (1.11%), andstigmast-5-en-3-ol (1.04%). The rest of the volatile constituents were found at concentrations lower than 1% (caryophyllene, hexadecanoate, 2H-furo[2H]-1-benzopyran-2-one, solanesol, and stigmasterol).

### 3.2. Nanosponge Fabrication

In the present study, the blank nanosponges were fabricated by using a melt method in five different molar concentrations (Table 1) using β-CD and DPC and their physico-chemical characterization was performed.

The molar ratio (β-CD: DPC) used in nanosponges was found to affect their practical yield. It has been observed that the practical yield increased with an increase in the molar ratio. This may be due to an increase in the number of reactive functional groups at higher concentrations.

### 3.3. Solubilization Efficiency of Nanosponges

**Figure 1:**
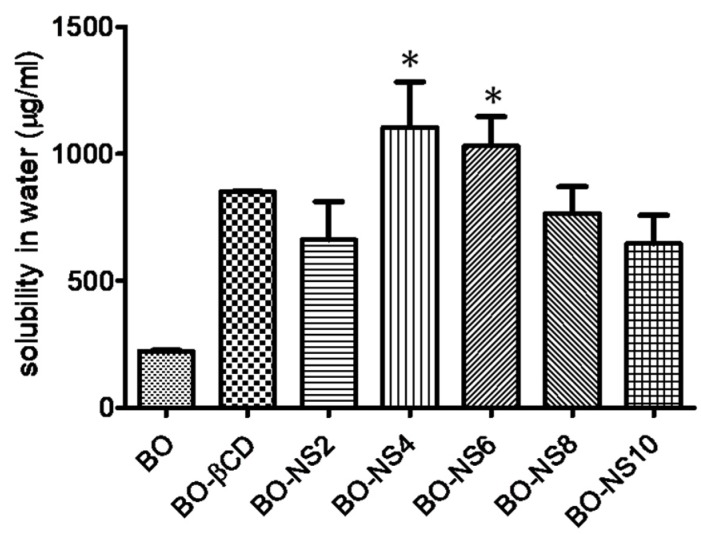
Solubilization of babchi oil (BO) by nanosponges (having different cross-linking density) and β-CD. Statistical data analysis from the one-way ANOVA (and nonparametric) Kruskal-Wallis test (solubilisation effeciency: Kruskal-Wallis statistics: 15.10) was followed by the Dunn’s multiple comparison test (* *p* < 0.05 versus BO).

The solubilization capacity of β-CD and all the prepared nanosponges for BO was evaluated and compared with the solubility of the free BO in double distilled water. All the five types of nanosponges (NS2–NS10) enhanced the solubility of the BO in comparison to free BO, which is shown in Figure 1 (Kruskal-Wallis statistics: 15.10 with *p* < 0.05). Among all, NS4 showed maximum solubilisation efficiency (1105 μg/mL) followed by NS6 (1030 μg/mL) in comparison with free BO (223.2 μg/mL). With plain β-CD, free BO exhibited solubilisation efficiency (851.1 μg/mL). In this paper, solubilisation efficiency data depicted the superiority of β-CD nanosponges over plain β-CD. The formation of the inclusion complex with the BO as well as encapsulation in the nanosponge matrix may have resulted in enhanced solubilization of this oil. This observation demonstrated that the encapsulation of BO in nanosponges resulted in the solubility enhancement of this essential oil. However, low solubilization efficiency of NS2, NS8, and NS10 was observed in comparison to plain β-CD for BO. In NS2, the degree of cross-linking may be low resulting in insufficient nanochannels, which do not have a remarkable impact on the solubility of free BO. However, in NS8 and NS10 formulations, a higher degree of cross-linking created more tortuous and complex nanochannels, which led to the prevention of the entrapment of BO into the NS structures. Anandum and Selvamuthukumar reported that the solubilization of quercetin in milli-Q water was enhanced by 20 times using β-CD and DPC as cross linker [19].

### 3.4. Loading and Encapsulation Efficiency

**Figure 2:**
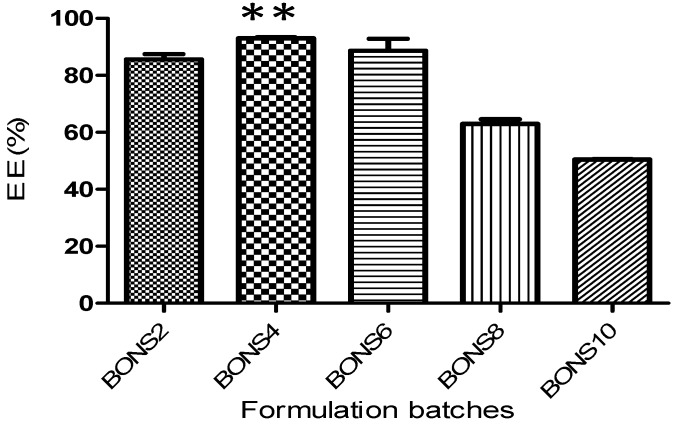
Encapsulation efficiency of the nanosponges (NS2–NS10). Statistical data analysis from a one-way ANOVA (and nonparametric) Kruskal-Wallis test (Encapsulation effeciency: Kruskal-Wallis statistics: 16.97) was followed by a Dunn’s multiple comparison test (** *p* < 0.01 versus BONS10).

BO was loaded into all five types of nanosponges by using the freeze drying technique. Figure 2 indicates loading efficacy of BO by NS in different molar ratios. Among the five types of nanosponges, the loading efficiency was observed to be higher in BONS4 (1:4) by as much as 21.47% *w*/*w* followed by BONS6 (20.44%) > BONS2 (19.75%) > BONS8 (14.53%) > BONS10 (14.23%). The results revealed that the degree of cross-linking affected the complexation efficiency of nanosponges. It was found that, at the 1:2 molar ratio, the degree of cross-linking may be low, which would result in insufficient nanochannels for the guest complexation. Thus, BO might not be encapsulated in higher amounts. While in BONS6, BONS8 and BONS10, which are the higher amount of the cross-linker resulted in hyper cross-linking of β-CD, ledto a hindrance in the interaction of BO with β-CD cavities. The encapsulation efficiencies of BONS formulations were found to be between 61% and 93% (Table 2).

The encapsulation efficiency of entire formulations followed the order: BONS4 ≥ BONS6 ≥ BONS2 ≥ BONS8 ≥ BONS10, which is shown in Figure 2. It was found that, for the babchi oil NS, the encapsulation efficiency was highest in BONS4 as much as 93% while being 61% in BONS10. The reason for more encapsulation of the BO in a 1:4 molar ratio may be due to optimum crosslinking involving inclusion and external interactions at the same time, which provides a higher quantity of oil to encapsulate in the nanosponge matrix as well as a cyclodextrin cavity. The effect of β-CD and a crosslinker ratio on entrapment efficiency of β-CD-based nanosponges has been extensively reported [37].

### 3.5. Particle Size, Polydispersity Index, and Zeta Potential Determination

The particle size of the BO nanosponges ranged from 234 to 484 nm, which is presented in Table 2. All the prepared BO nanosponges depicted particle size in the nano range (<1 μm). Zeta potential of different BO nanoformulations was also checked as a measure of surface charge. The results of zeta potential obtained are presented in Table 2. High zeta potential shows that the nanosponge would be stable due to a higher magnitude of repulsive forces, which leads to a reduction in their tendency to aggregate. Reduced values of PDI with a narrow range showed that the nanocolloidal suspensions are stable and homogenous in nature. All the prepared nanoformulations were found as fine and free-flowing powders.

Based on the results of the encapsulation and solubilization efficiency, formulation BONS4 was selected for further characterization through FTIR, TGA, XRPD, FE-SEM, and TEM.

### 3.6. Fourier Transform Infrared Spectroscopy

The BO, blank nanosponges, and BONS4 were subjected to FTIR analysis. The FTIR spectrum of the BO showed characteristic absorption bands at around 3434, 2927, 2855, 1744, 1622, 1458, 1376, 1240, 1169, and 724 cm^−1^.

Plain NS exhibited a characteristic peak around 1777 cm^−1^ due to a carbonate bond and 3368 cm^−1^ due to O–H stretching vibration, which confirms the formation of CD-based nanosponges. Additionally, other prominent peaks of NS were found at 2926 cm^−1^ due to a C–H stretching vibration, 1419 cm^−1^ due to a C–H bending vibration, and 1029 cm^-1^ due to a C–O stretching vibration of primary alcohol. FTIR spectrum of β-CD (starting material for NS synthesis) showed no peak around 1700 cm^−1^. Hence, this ascertains the formation of a carbonate bond after their interaction with cross-linker (DPC) in nanosponges.

**Figure 3:**
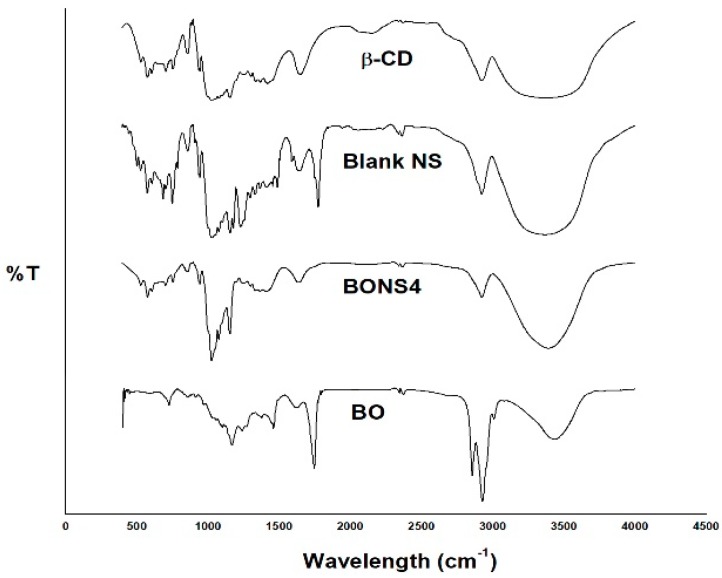
FTIR spectra of BO, β-CD, blank NS, and BONS4.

The comparison of FTIR spectra of BO, blank NS, and BONS4 evidenced that the characteristic peaks of the BO were broadened or shifted in nanoformulations, which suggests interactions between oil and nanosponges (Figure 3).

### 3.7. Thermal Analysis

**Figure 4:**
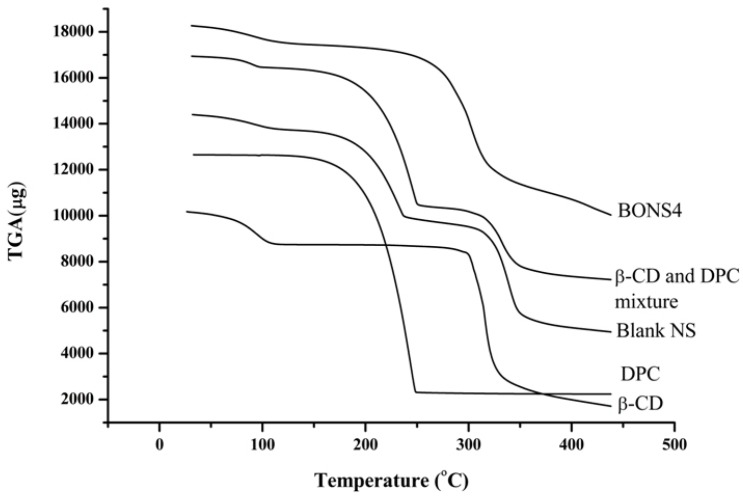
TGA curves of β-CD, DPC, blank NS, β-CD and DPC mixture, and BONS4.

**Figure 5:**
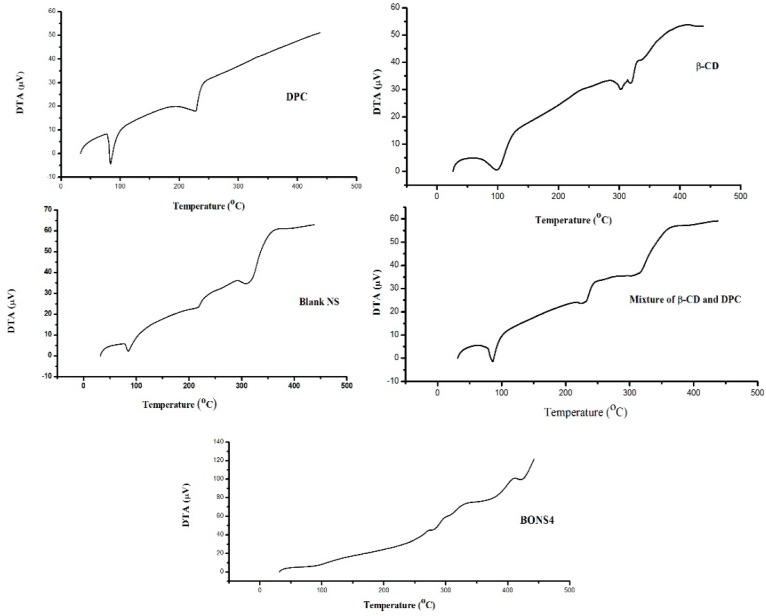
DTA curves of β-CD, DPC, blank NS, mixture of β-CD and DPC, and BONS4.

Thermo gravimetry analysis is an efficient way to examine alterations in chemical and physical properties of materials. Thermal analysis of β-CD, DPC, blank NS, and their complexes ascertained not only a presence of an inclusion compound but also a physical mixture of β-CD and DPC. Figure 4 and Figure 5 depicted the results of TGA and DTA for β-CD, DPC, blank NS, and BONS4, respectively.

On one hand, pure β-CD suffers a first weight loss (13.8%) at 112 °C, which is related to the evaporation of water associated with a polymer (β-CD). The second zone around 300 °C to 337 °C with 53.7% weight loss can be attributed to the degradation of the β-CD. The third zone of weight loss (27.9% to 16.8%) from 337 °C to 438 °C can be assigned to char degradation. On the other hand, DPC showed weight loss (96.2%) at 229 °C, which indicates its complete degradation. In case of the blank nanosponges, the first weight loss (6.0%) was observed at 100 °C, which corresponds to water evaporation followed by second weight loss (35.8%) at 100°C to 218 °C and finally a third weight loss (37.0%) indicated at the 289 °C to 338 °C. A slight shift of peaks was observed in the case of BO nanosponges, which is observed in the blank nanosponges.

The TGA curve of the β-CD and DPC mixture showed some similarity with blank nanosponges, which ascertained some degree of crosslinking between the polymer and DPC with an increase in temperature. The peak below 100 °C is associated with a residual moisture in the respective sample. However, for all nanosponges, the maximum degradation processes of their cross linked structures occur at 240 to 300 °C, which indicates good thermal stability. DTG results helped in further strengthening the TGA findings (Table 3).

**Figure 6:**
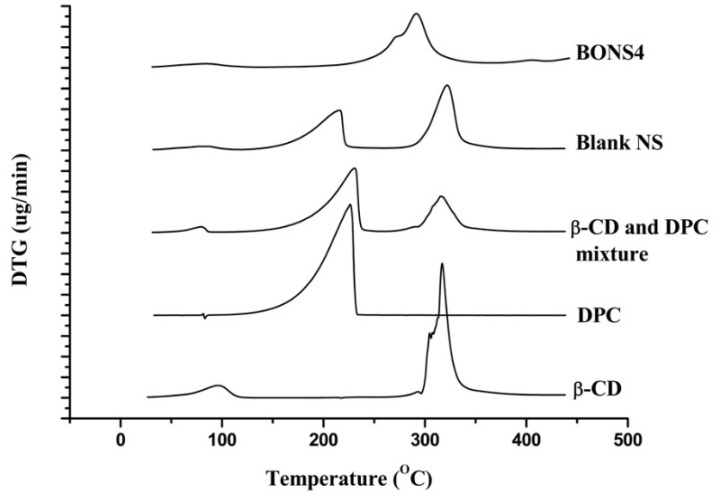
DTG curves of β-CD, DPC, β-CD and DPC mixture, blank NS, and BONS4.

Differential thermal analysis measures the temperature difference between the sample and the reference, which resulted in heat absorption or liberation. As shown in Figure 6, the absence of a characteristic peak in BONS4 indicated the change of the physical state of the components in nanosponges. As indicated in Table 4, these results corroborate the previous findings of thermal analysis of the nanosponges.

### 3.8. X-Ray Powder Diffraction

**Figure 7:**
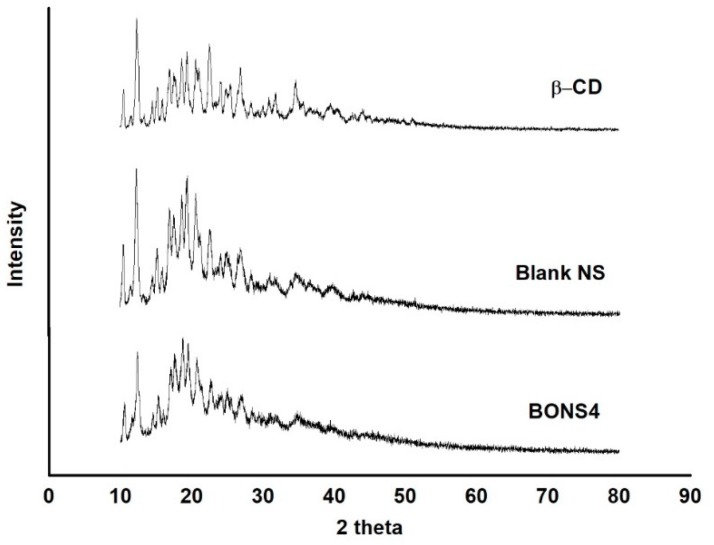
XRPD patterns of β-CD, blank NS, and BONS4.

The diffraction pattern of blank nanosponges and BONS4 has been shown in Figure 7. A significant difference between their diffractogram exists, i.e., reduction in the intensity of peaks was observed. Hence, the XRPD studies indicated that, after freeze drying (BONS4), a fluffy powder was obtained with a highly porous structure losing its crystallinity. The characteristic peaks of the blank nanosponges were observed at 10.53°, 12.37°, 15.14°, 16.99°, 18.60°, 19.29°, 20.92°, 22.52°, 24.15°, 25.30°, 26.90°, 28.52°, 31.05°, 34.75°, 36.60°, and 39.83° (2θ), which is indicated in Figure 7. The intensity of these peaks were weakened in the BO loaded nanosponges (Figure 7), which depicts a reduction of the crystallinity of the CD nanosponges after encapsulation of BO.

### 3.9. Surface Morphology

**Figure 8:**
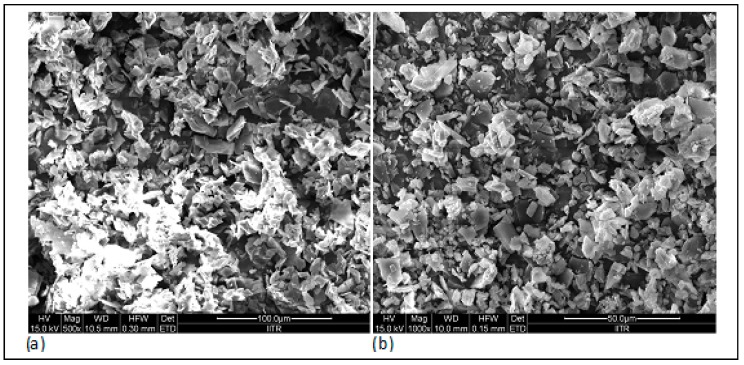
Field emission scanning electron microscopy: (**a**) plain NS and (**b**) BONS4.

**Figure 9:**
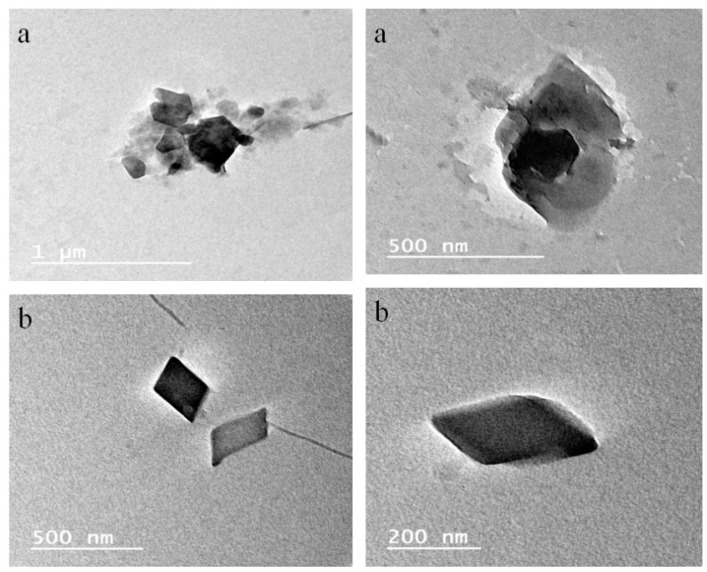
TEM images of (**a**) blank nanosponges and (**b**) BONS4.

The surface topography of the prepared nanosponges was also studied using scanning electron microscopy. As observed in FE-SEM images (Figure 8), cyclodextrin nanosponges showed crystalline morphologies. Additionally, the morphology of the selected BO nanosponges (BONS4) and blank nanosponges was studied by using transmission electron microscopy. Using TEM, a single crystal of a nanosponge can be focused by elucidating its definite crystalline geometry. As shown in representative TEM photographs (Figure 9), the dimensions of the crystal lattice agreed well with XRPD findings. The morphological characterization by this microscopy showed that the nanosponges prepared by a melt method, possesses a uniform size distribution, crystallinity, and a porous nature. Furthermore, lattice planes lying straight across the nanosponges indicated that the obtained nanosponges possess a high degree of crystallinity.

### 3.10. Cytotoxicity Studies against HaCaT Cell Lines

Most of the essential oils have been reported to produce toxicity or skin irritation in spite of their good topical advantages. To compare the cytotoxicity of plain BO and BONS4, the dose response curve was established. The results indicated that cytotoxicity of the BONS was slightly less than that of BO. Hence, in order to explore the potential benefits of BO loaded nanosponges on human keratinocytes, the cytotoxicity of BO and BO loaded nanosponges were studied using the MTT assay and the results of human skin cells treated with different concentrations are presented in Table 5.

Both of these samples resulted in dose-dependent reductions in cellular viability. Furthermore, the MTT assay showed that the treatment of these cells with BO loaded nanosponges at 320 μg/mL resulted in a cytotoxic effect with IC50 value 191.4 μg/mL and plain BO with an IC50 value of 172.3 μg/mL. However, there is no significant difference between the cytotoxicity caused by BO and BONS, which indicates that a sufficient quantity of the drug is released from nanosponges to cause cell toxicity. Hence, the results of the MTT assay indicated that the developed nanoformulation is safer on human skin cells in comparison to babchi essential oil (Table 5).

### 3.11. Photodegradation Study

BO gets absorbed in the UV region that exhibits a peak around 265 nm. The intensity of this peak was found to be diminished upon UVA illumination, which ascertains photolysis of BO constituents.

With the objective of evaluating the protective phenomenon of nanosponges on the photo degradation of the BO constituents, the photo degradation kinetics of this oil and oil-loaded nanosponges was also compared [18,38,39].

The BO concentration was plotted as a function of irradiation time to analyze the photo degradation kinetics. The obtained exponential asymptotic curve of the degraded BO upon BO irradiation followed first order kinetics. The following logarithmic equation was used for the calculation of the slope of the obtained curves.

$$\ln C_{t}/C_{0}=-k\times t$$

where *C*_0_ is the initial concentration of BO and *C_t_* represents its residual concentration at time *t*. The rate constant data can be calculated using the linear dependence of ln*C_t_*/*C*_0_ vs. time. The *k* values were calculated using the pseudo-first order model fitting (Table 6).

A comparison of rate constants of photo degradation exhibited by BO (6.909 × 10^−3^ ± 0.63 min^−1^) and BO loaded nanosponges (2.303 × 10^−3^ ± 0.47 min^−1^) advocated that BO loaded nanosponges are capable of slowing the photo oxidation process due to the physical barrier provided by nanosponges against UV-induced oxidation of BO.

### 3.12. Antibacterial Activity

**Figure 10:**
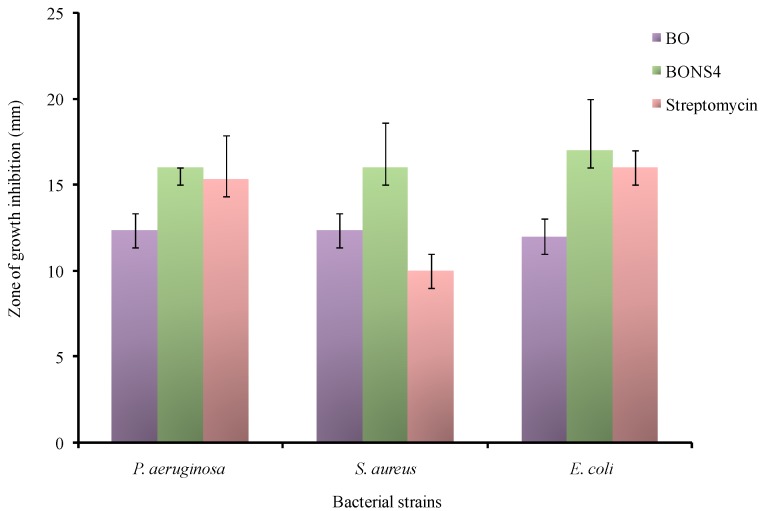
Antibacterial activities of *Psoralea corylifolia* seed oil. BO-Babchi oil and BONS4-Optimized babchi oil loaded nanosponges.

BO loaded nanosponges showed a clear inhibitory effect against *P. aeruginosa*, *E. coli*, and *S. aureus.* Yet, babchi essential oils also exhibited an inhibitory effect. The antibacterial effect was remarkably improved for BO-loaded nanosponges. The zone of growth inhibition for BO were as follows: 12.33 ± 2.5 mm for *S. aureus*, 12 ± 2.4 mm for *E. coli*, 12.33 ± 2.3 mm for *P. aeruginosa* while, in BONS dispersion, it was 16.00 ± 2.64 mm for *S. aureus*, 17 ± 3.00 mm for *E. coli*, and 16 ± 0.00 mm for *P. aeruginosa* (Figure 10). The positive control streptomycin (10 μg/mL) produced a comparable zone of growth inhibition against *S. Aureus* (10.00 ± 1.00 mm), *E. coli* (16 ± 1.00 mm for), and *P. aeruginosa* (15.33 ± 2.51 mm) while negative control (blank nanosponge dispersion) plates did not show growth inhibition of the above mentioned bacteria. The restraining antibacterial effects of BO against *P. aeruginosa*, *E. coli*, and *S. aureus* can be assigned due to water insolubility and the volatile nature of oil. As observed from solubility studies, the entrapment of the BO in cyclodextrin nanosponges potentially improved the water solubility, which might have resulted in the enhancement of antibacterial activity.

## 4. Conclusions

The present study reports on the encapsulation of babchi essential oil in β-cyclodextrin nanosponges. First, GCMS analysis of BO was performed in order to identify the compounds present in this oil. A solubilization efficiency assessment showed all five types of fabricated nanosponges, which enhanced the solubility of BO in comparison to free BO. The BO was efficiently loaded in diphenylcarbonate cross-linked β-cyclodextrin nanosponges using a freeze drying technique. The results of the encapsulation efficiency implies that an optimum ratio of the polymer to the cross linker in BO nanosponges is 1:4 (molar ratio) and the product processed from this molar ratio can help deliver a therapeutically effective dose. Spectral characterization data revealed the formation of stable inclusion complexes in nanosponges. The prepared nanosponges displayed good thermal stability. Cytotoxicity studies explored the enhancement in the therapeutic response that will retard the overall drug consumption and dose and will minimize systemic side effects due to drug localization at the target site. On the basis of calculated kinetic parameters, the nanosponge complexation minimizes the UV photodegradation. The fabricated optimized formulation was also found active on bacteria such as *P. aeruginosa, E. coli*, and *S. aureus* when compared with pure essential oil. Furthermore, research on the pharmacological evaluation of BO nanosponges is in progress. It will be helpful to explain the potential of BO nanosponges for therapeutic applications.

## Figures and Tables

**Table 1:** Molar ratios of β-cyclodextrin and diphenyl carbonate (*n* = 3, mean ± SD).

Sr. No.	Nanosponge Type	Molar Ratio β-CD: DPC	Amount of β-CD (g)	Amount of DPC (g)	Practical Yield (g) ± SD
1	NS2	1:2	4.548	1.712	3.544 ± 0.220
2	NS4	1:4	4.548	3.424	4.559 ± 0.199
3	NS6	1:6	4.548	5.136	5.8966 ± 0.197
4	NS8	1:8	4.548	6.848	6.434 ± 0.197
5	NS10	1:10	4.548	8.56	6.735 ± 0.296 *

Statistical data analysis from the one-way ANOVA (and nonparametric) Kruskal-Wallis test (practical yield: Kruskal-Wallis statistics: 13.23) was followed by the Dunn’s multiple comparison test (* *p* < 0.05 versus NS2).

**Table 2:** Particle sizes, zeta potentials, poly dispersity index, and percentage encapsulation efficiency of the BO formulations (*n* = 3, mean ± SD).

Sr. No.	Formulation	Particle Size (nm ± SD)	Zeta Potential (mV ± SD)	Poly Dispersity Index ± SD	% Encapsulation Efficiency ± SD
1.	BONS1:2	261.6 ± 14.79	−17.8 ± 2.52	0.312 ± 0.098	85.61 ± 1.848
2.	BONS1:4	360.9 ± 11.55	−16.0 ± 1.15	0.311 ± 0.059	93.05 ± 0.283 **
3.	BONS1:6	234.3 ± 15.37	−15.5 ± 1.17	0.188 ± 0.064	88.61 ± 4.286
4.	BONS1:8	484.2 ± 19.89	−15.6 ± 2.39	0.509 ± 0.236	62.98 ± 1.669
5.	BONS1:10	243.3 ± 12.95	−22.0 ± 2.47	0.361 ± 0.113	50.43 ± 0.173

Statistical analysis fromthe one-way ANOVA (and nonparametric) Kruskal-Wallis test (encapsulation efficiency: Kruskal-Wallis statistics: 16.97) followed by the Dunn’s multiple comparison test. ** *p* < 0.01 versus BONS10.

**Table 3:** Derivative thermogravimetric analysis of the fabricating materials and nanosponges.

Formulating Materials and NS	Derivative Thermo Gravimetric Parameters		
	Temperature (°C)	Type of Peak	Derivative of Thermo Gravimetry (mg/min)
β-CD	98	Exothermic	0.30
	304	Exothermic	1.54
	317	Exothermic	3.26
DPC	226	Sharp exothermic	2.69
β-CD and DPC mixture	80	Exothermic	0.14
	231	Sharp exothermic	1.57
	315	Exothermic	0.88
Blank NS	217	Exothermic	0.96
	323	Exothermic	1.57
BONS4	272	Exothermic	0.76
	291	Exothermic	1.31

**Table 4:** Differential thermal analysis of the fabricating materials and nanosponges.

Formulating Materials and NS	Parameters of Differential Thermal Analysis			
	Temperature (°C)	Type of Peak	Change in Enthalpy (∆H) mJ/mg	DTA Sensitivity (μV)
β-CD	98	Endothermic	224	0.6
	302	Endothermic	110	30
	320	Endothermic	110	32.51
DPC	83	Endothermic	96.2	−4.30
	228	Endothermic	146	17.88
β-CD and DPC mixture	86	Endothermic	108	−1.50
	231	Endothermic	49.5	24.3
	317	Endothermic	160	37
Blank NS	85	Endothermic	36.6	2.3
	218	Endothermic	35.3	23.4
	314	Endothermic	189	35.3
BONS4	-	-	-	-

**Table 5:** Effect of BO and BONS on the viability of HaCaT cells as a function of drug concentration at 24 h.

Sample	Concentration µg/mL	% Inhibition ± Standard Deviation (*n* = 3)	IC50
Control	0	0.00 ± 0.00	172.3 µg/mL
Babchi oil (BO)	10	4.41 ± 1.21	
	20	7.48 ± 1.47	
	40	16.84 ± 1.40	
	80	23.45 ± 1.20	
	160	38.61 ± 2.16	
	320	51.16 ± 2.23	
Babchi oil loaded nanosponges (BONS)	10	4.26 ± 1.96	191.4 µg/mL
	20	9.60 ± 1.63	
	40	16.39 ± 1.74	
	80	25.86 ± 2.29	
	160	48.73 ± 2.09	
	320	61.27 ± 1.06	

**Table 6:** Correlation coefficient (*r*^2^) and rate constant (*k*) of BO and BONS4 photo degradation under UVA irradiation.

Sr. No.	Oil and Its Formulation	Correlation Coefficient (*r*^2^)	Rate Constant (*k*) (1 × 10^−3^ min^−1^)
1.	BO	0.953 ± 0.14	6.909 ± 0.63
2.	BONS4	0.405 ± 0.10	2.303 ± 0.47
